# Fetal growth trajectories in pregnancies of European and South Asian mothers with and without gestational diabetes, a population-based cohort study

**DOI:** 10.1371/journal.pone.0172946

**Published:** 2017-03-02

**Authors:** Line Sletner, Anne Karen Jenum, Chittaranjan S. Yajnik, Kjersti Mørkrid, Britt Nakstad, Odd Harald Rognerud-Jensen, Kåre I. Birkeland, Siri Vangen

**Affiliations:** 1 Dept. of Pediatric and Adolescents Medicine, Akershus University Hospital, Lørenskog, Norway; 2 Dept. of General Practice, Institute of Health and Society, University of Oslo, Oslo, Norway; 3 Diabetes Unit, King Edward Memorial Hospital Research Centre, Pune, India; 4 Dept. of International Public Health, Norwegian Institute of Public Health, Oslo, Norway; 5 Institute of Clinical Medicine, Campus Ahus, University of Oslo, Lørenskog, Norway; 6 Faculty of Health, Oslo and Akershus University College of Applied Sciences, Oslo, Norway; 7 Department of Endocrinology, Morbid Obesity and Preventive Medicine, Oslo University Hospital, Oslo, Norway; 8 Institute of Clinical Medicine, University of Oslo, Oslo, Norway; 9 Norwegian National Advisory Unit on Women`s Health, Department for Woman and Child Health, Oslo University Hospital, Oslo, Norway; Holbæk Hospital, DENMARK

## Abstract

**Objective:**

Our aim was to examine the impact of gestational diabetes (GDM), from before the GDM-diagnosis is made, on fetal growth trajectories, and to compare it in Europeans and South Asians; two ethnic groups with dissimilar fetal growth patterns.

**Methods:**

We studied European (n = 349) and South Asian (n = 184) pregnant women, from the population-based STORK-Groruddalen cohort in Oslo, Norway. Mothers were enrolled in early pregnancy, screened for GDM in gestational week 28 ±2, and classified as “non-GDM”, “mild GDM” or “moderate/severe GDM”. We measured fetal head circumference, abdominal circumference and femur length by ultrasound, and estimated fetal weight in gestational week 24, 32 and 37, and performed corresponding measurements at birth.

**Results:**

In non-GDM pregnancies, South Asian fetuses (n = 156) had a slower growth from gestational week 24, compared with Europeans (n = 310). More than two thirds of the European mothers later diagnosed with GDM were overweight or obese in early pregnancy, while this was not observed in South Asians. Fetuses of GDM mothers tended to be smaller than fetuses of non-GDM mothers in week 24, but thereafter grew faster until birth. This pattern was especially pronounced in fetuses of South Asian mothers with moderate/severe GDM. In week 24 these fetuses had a -0.95 SD (95% CI: -1.53, -0.36) lower estimated fetal weight than their non-GDM counterparts. In contrast, at birth they were 0.45 SD (0.09, 0.81) larger.

**Conclusions:**

Offspring of GDM mothers were smaller in mid pregnancy, but subsequently grew faster until birth, compared with offspring of non-GDM mothers. This pattern was most prominent in South Asian mothers with moderate to severe GDM. However, the most remarkable characteristic of these fetuses was not a large size at birth, but the small size in mid pregnancy, before the GDM diagnosis was set.

## Introduction

The association between birth weight and later risk of type 2 diabetes is U-shaped. One arm comprise men and women born small, due to maternal undernutrition or other factors. The other arm comprises of individuals born large, due to maternal adiposity and diabetes [1].

South Asians have a higher prevalence of type 2 diabetes compared with Europeans, and are diagnosed at a younger age and with a lower BMI [2]. The underlying mechanisms are only partly understood, but probably include that South Asian babies are born smaller, but relatively adipose, i.e. have a reduced lean mass, but higher fat mass, compared with Europeans. This “thin-fat” phenotype of South Asians tracks through life, and could result in increased insulin resistance [3]. South Asian pregnancies present a mixture of characteristics: on one hand shorter maternal height, lower BMI and a higher risk of micro-nutritional deficiencies; factors which may constrain fetal growth, on the other hand higher adiposity, insulin resistance and hyperglycemia which may enhance growth [4, 5].

The usual description of a baby born to a mother with gestational diabetes (GDM) includes macrosomia and large for gestational age phenotypes in late pregnancy and at birth. Some studies indicate a stronger association between maternal hyperglycemia and offspring birth weight in ethnic South Asians than in Europeans [6–8]. However, little is known about the fetal growth trajectories in GDM and non-GDM pregnancies, in populations universally screened for GDM, and if this differs between these two ethnic groups.

The population-based multi-ethnic STORK-Groruddalen cohort provided a unique opportunity to explore these relationships. We compared maternal characteristics in early pregnancy, and fetal size and growth rate during the second half of pregnancy, in ethnic South Asian and European pregnancies with and without GDM in Oslo, Norway.

### Ethics statement

Pregnant women attending the Child Health Clinics for antenatal care in three municipalities in Groruddalen, Oslo, from May 2008 to May 2010 were given oral and written information about the Stork Groruddalen project and invited to participate. The women who chose to participate gave informed written consent at inclusion, on behalf of themselves and their offspring [9]. The study protocol and the consent-forms were approved by The Regional Committee for Medical and Health Research Ethics for South Eastern Norway, and The Norwegian Data Inspectorate.

### Population and design

The study design has been presented in detail elsewhere [9]. Prior to the study, 75–85% of all pregnant women in this area attended the local Child health Clinics during their pregnancy. Questionnaire data was collected by specially trained study midwives through interview, supported by a professional interpreter, using translated questionnaires when needed. Information material and questionnaires were translated to eight languages including English, Tamil and Urdu, covering the largest South Asian ethnic groups. Women were eligible if they were: (1) living in one of the districts, (2) would give birth at the study hospitals, (3) were in gestational week < 20, (4) not suffering, at the time of enrollment, from diseases necessitating intensive hospital follow-up during pregnancy (i.e. pre-gestational diabetes and other substantial medical, psychiatric or obstetrical conditions) (5) not already enrolled with a pregnancy lasting > 22 weeks, (6) could communicate in Norwegian or any of the other eight languages, and (7) were able to give informed consent. The final sample consisted of 823 pregnant women. Overall participation rate was 74% (81.5% in Europeans and 73.0% in South Asians) and the study cohort was considered representative for women attending the Child Health Clinics with respect to ethnicity and age [9].

### Maternal factors

The participating woman and fetus were defined as ethnic European or South Asian if the participating woman or her mother were born in Europe or South Asia respectively. Europeans also included three women of European origin born in North-America [10]. As few South Asians migrated to Norway before the 1970`s, all potential participants with South Asian ancestry were either born in South Asia, or their mothers were born there.

Glycated haemoglobin (HbA1c) and fasting glucose were measured at enrollment. HbA1c was measured with HPLC (Tosoh G8,Tosoh Corporation, normal reference range 4–6%). A standard 75 g oral glucose tolerance test (OGTT) was performed in gestational week 28±2. Women were diagnosed with gestational diabetes according to the 1999 WHO-criteria (fasting plasma glucose ≥7.0 mmol/l or 2-hour glucose ≥7.8 mmol/l). Glucose was measured on site (within 5 minutes after vein puncture) in venous EDTA blood according to a standardized protocol, using a patient-near method (HemoCue 201+, Angelholm Sweden) calibrated for plasma [9, 11]. According to national guidelines, women with fasting glucose < 7.0 mmol/l and 2-hour values 7.8–8.9 mmol/l were categorized as “mild GDM”, and were given oral and written lifestyle advice and referred to their general practitioner for follow-up [9]. Women with fasting glucose ≥ 7.0 mmol/l or 2-hour values ≥ 9.0 mmol/l were categorized as “moderate/severe GDM”, and referred to secondary care. Data related to GDM-treatment have been collected from hospital records.

Maternal height was measured twice to the nearest 0.1 cm with a fixed stadiometer at inclusion, and the mean was used [9]. Pre-pregnant body mass index (BMI, kg/m^2^) was calculated using self-reported pre-pregnant weight, to the nearest kg, and measured height [9]. Weight gain (WG) in kg, was calculated from pre-pregnancy to enrollment, from enrollment to week 28 and from week 28 to birth (see footnotes Tables 1 and 2). Parity was categorized as “primiparous” or “parous” (at least one previous pregnancy lasting > 22 weeks). Except in 7% of women gestational age was derived from the first day of the woman’s last menstrual period (see footnote Table 1).

**Table 1:** Maternal characteristics by ethnic origin.

	Europe	South Asia	
	n = 349	n = 184	p
Sociodemographics			
Age, years	30.6 (4.5)	28.3 (4.5)	<0.001
Primiparous	185 (53%)	76 (42%)	0.01
Educational level			<0.001
<10 years	15 (4%)	33 (18%)	
High school	109 (32%)	91 (50%)	
College/university	222 (64%)	59 (32%)	
Married/cohabitant	334 (96%)	182 (99%)	0.05
Born and raised in Norway^a^	288 (83%)	23 (13%)	<0.001
Smoking at inclusion^b^	26 (7%)	1 (<1%)	0.001
Gestational age at inclusion^c^	14.6 (2.4)	15.9 (4.2)	<0.001
Anthropometry			
Height, cm	167.2 (5.7)	160.0 (5.6)	<0.001
Pre-pregnant BMI, kg/m^2^	24.6 (4.8)	23.7 (4.1)	0.06
Overweight (pre-pregnant BMI 25–29.9 kg/m^2^)	82 (24%)	40 (22%)	0.6
Obesity (pre-pregnant BMI ≥ 30 kg/m^2^)	45 (13%)	17 (9%)	0.2
BMI at inclusion, kg/m^2^	25.2 (4.8)	24.4 (4.1)	0.05
Sum of skinfolds (mm)	69.4 (19.8)	74.4 (18.9)	0.008
Glucose parameters at inclusion			
Fasting glucose (mmol/l)	4.4 (0.4)	4.5 (0.4)	0.03
HbA1c, %/mmol/mol)	5.1 (0.2)/32	5.2 (0.3)/33	<0.001
Glucose parameters in week 28			
Fasting glucose (mmol/l)	4.7 (0.6)	4.9 (0.6)	<0.001
2-hour glucose, mmol/l	6.0 (1.4)	6.4 (1.5)	0.003
Number of ultrasound study measurements			<0.001
1	6 (2%)	12 (7%)	
2	50 (14%)	57 (31%)	
3	293 (84%)	115 (63%)	

Numbers are n (%) or mean (SD), and the p-value indicates the level of significance for the difference between the two ethnic groups, using t-tests for continuous and chi-square tests for categorical variables.

^a^ Born in Norway and lived more than eight of her sixteen first years in Norway.

^b^ Daily or occasional smoking

^c^ Gestational age was derived from the first day of the woman’s last menstrual period (LMP). Term was calculated as date of LMP + 282 days. In 7% of women (in both ethnic groups) LMP date was either unknown/uncertain, LMP derived term differed >14 days from ultrasound term (from week 18–20 routine scan) or the pregnancy was a result of in vitro fertilization. Ultrasound term was used in these cases.

**Table 2:** Maternal characteristics by ethnic group, and differences between women diagnosed with mild or moderate/severe GDM, compared with non-GDM women.

		Europe			South Asia	
	non-GDM (ref)	Mild	Moderate/severe	non-GDM (ref)	Mild	Moderate/severe
	n = 310	n = 30	n = 9	n = 156	n = 14	n = 14
Sociodemographics						
Age, years, mean (95% CI)	30.6 (30.1, 31.1)	31.2 (29.5)	30.6 (27.6, 33.5)	28.4 (27.7, 29.1)	30.7 (28.3, 33.0)	30.4 (28.0, 32.7)
Primipara	149 (48)	10 (33)	5 (56)	63 (40)	6 (43)	7 (50)
Educational level						
Primary school	13 (4)	2 (7)	0 (0)	26 (17)	3 (21)	4 (29)
10–12 high school education	98 (32)	8 (27)	3 (33)	79 (51)	6 (43)	7 (43)
College/university education	196 (64)	20 (67)	6 (67)	50 (32)	5 (36)	4 (29)
Born and raised in Norway^a^	258 (83)	23 (77)	7 (78)	21 (14)	2 (14)	0 (0)
Smoking at enrollment^b^	24 (8)	1 (3)	1 (11)	1 (1)	0 (0)	0 (0)
Gestational age at enrollment, mean (SD)	14.2 (13.9, 14.5)	14.1 (13.3, 14.9)	14.7 (13.1, 16.2)	15.4 (14.8, 16.0)	16.1 (14.0, 18.1)	17.9 (15.8, 20.0)*
Pre-pregnant anthropometrics						
Height, mean (95% CI)	168 (167, 169)	164 (162,166)*	165 (161, 169)	160 (159, 161)	159 (156, 162)	158 (155, 161)
BMI, kg/m^2^	24.3 (23.8, 24.8)	25.5 (23.8, 27.2)	30.5 (27.4, 33.6)*	23.7 (23.0, 24.3)	25.3 (23.2, 27.5)	22.7 (20.6, 24.9)
Overweight, BMI 25–29.9 kg/m^2^	67 (22)	13 (43)*	2 (22)	32 (21)	6 (43)	2 (14)
Obesity, BMI ≥ 30 kg/m^2^	34 (11)	5 (17)	6 (67)***	15 (10)	1 (7)	1 (7)
Biomarkers at enrollment						
Fasting glucose, mmol/l, mean (95% CI)	4.4 (4.3, 4.4)	4.5 (4.4, 4.7)*	4.7 (4.5, 5.0)**	4.5 (4.4, 4.5)	4.6 (4.4, 4.8)	4.6 (4.4, 4.9)
Fasting glucose > 5.1 mmol/l	11 (4)	1 (3)	1 (11)	11 (7)	3 (21)	2 (15)
HbA1c, %, mean (95% CI)^c^	5.1 (5.0, 5.2)	5.2 (5.1, 5.2)	5.3 (5.1, 5.4)	5.2 (5.2, 5.3)	5.4 (5.3, 5.6)*	5.3 (5.1, 5.4)
HbA1c ≥ 5.7%(39 mmol/mol)	3 (1)	2 (7)	0 (0)	7 (5)	2 (14)	1 (7)
Weight gain (WG)						
WG, kg, pre-preg to inclusion^d^	2.1 (1.7, 2.5)	1.3 (0.1, 2.4)	-1.2 (-3.4, 0.9)**	1.6 (1.0, 2.2)	1.4 (-0.5, 3.4)	2.5 (0.6, 4.4)
WG, kg, inclusion to week 28^e^	7.1 (6.8, 7.4)	6.2 (5.2, 7.2)	5.2 (3.4, 7.1)*	6.6 (6.0, 7.1)	5.6 (3.9, 7.4)	6.5 (4.7, 8.2)
WG, kg, week 28 to birth^f^	5.9 (5.5, 6.4)	4.0 (2.6, 5.5)**	2.0 (-0.4, 4.4)**	5.2 (4.5, 5.9)	5.1 (2.9, 7.4)	4.8 (2.5, 7.0)

Differences between GDM, compared with non-GDM women, within ethnic group, were assessed using chi-square tests for categorical and t-tests for continuous variables.

Numbers are n (%) or mean (95% CI).

* Indicates a significant difference from non-GDM women within ethnic group of p≤0.05

** Indicates a significant difference from non-GDM women within ethnic group of p<0.01

*** Indicates a significant difference from non-GDM women within ethnic group of p<0.001

^a^ Born in Norway and lived more than eight of her sixteen first years in Norway.

^b^ Daily or occasional smoking

^c^ HbA1converted to mmol/mol: 5.1% = 32 mmol/mol, 5.2% = 33 mmol/mol, 5.3% = 34 mmol/mol and 5.4% = 36 mmol/mol

^d^ Calculated from measured weight at inclusion and the self-reported pre-pregnant weight

^e^ Calculated from measured weight at each time point

^f^ Calculated as the difference between the total WG reported at the postnatal visit and measured weight in week 28. Women diagnosed with GDM were especially encouraged to attend the post-partum visit. Information about total weight gain is missing in 16% of non-GDM women, due to lower attendance rates.

### Fetal- and neonatal measurements

At each ultrasound visit, scheduled at 24, 32 and 37 weeks of gestation, participants were randomly allocated to one of four study-ultrasonographers. One Voluson Pro (GE-Healthcare) machine, with a AB2-7 scan head, was used. Methods have been described in detail elsewhere [12]. Using standard anatomical landmarks, abdominal circumference (AC) and head circumference (HC) were obtained by applying computer-generated elliptical measurements to the outer surfaces, while femur length (FL) was obtained in a longitudinal section. Each biometric variable was measured three times, according to a study-specific standardized protocol, and the mean value was used. Estimated fetal weight (EFW) was calculated using Combs formula: EFW = (0.23718 x AC^2^ x femur length) + (0.03312 x HC^3^) [13], as used in clinical practice in the study-hospitals at that time.

Birth weight was routinely measured on calibrated electronic scales immediately after birth. Within 72 hours after birth, study-specific anthropometric measurements were performed by trained study personnel, according to study protocol, unless contraindicated for medical reasons [9, 14]. Crown-heel-length (CH-length) was measured by a measuring rod, with the head firmly held, while stretching the legs. For circumference measurements a non-elastic plastic tape was used. CH-length, HC and AC were measured to the nearest 0.1 cm [14].

### Statistical methods

Z-scores for fetal measurements (differences in standard deviations from gestational age specific mean) were calculated using formulas derived from an ethnic Norwegian reference population [15, 16]. National birth weight references were used to calculate birth weight z-scores in the total study sample [17]. In the large sub-sample of term neonates with study-specific anthropometric measurements at birth, we have previously calculated individual z-scores, stratified by gestational week and gender for birth weight, HC, AC and CH-length [14].

Statistical analyses were performed using SPSS version 22.0 for Windows (SPSS Inc., Chicago, IL, USA). The significance level was set to p<0.05. Differences between pregnancies complicated by mild or moderate/severe GDM, compared with non-GDM pregnancies were explored using Chi Square test for categorical and t-tests for continuous variables.

Differences in fetal growth from week 24 until birth were examined using linear mixed effects modelling, assessed separately for the outcomes weight, HC, AC and length. Z-scores through four different time points (week 24, 32, 37 and at birth) were entered. For the analysis of length we used z-scores for femur length at three time points in pregnancy and CH-length from birth. For the analyses of weight we used z-scores of EFW from three time points during pregnancy and birth weight. All models were run with a random intercept and a random slope. We first explored the impact of ethnicity on fetal growth in non-GDM pregnancies. Ethnicity was first entered as a fixed variable together with gestational age (weeks) and the interaction term between these two variables (to assess the impact of ethnicity on growth velocity). A priori, we added the covariates offspring gender and maternal parity to the adjusted models. As lower maternal height is often perceived to explain a substantial part of ethnic differences in fetal growth, maternal height was also included in the last supplementary model. A second degree term for gestational age was also included as a fixed variable in all models, due to a small, but systematic variation in the distribution of the fetal z-scores across the four time points. Interaction terms between other covariates and gestational age were tested for and included if significant. We then performed similar analyses replacing ethnicity with GDM as explanatory variable, first treated as non-GDM or GDM, thereafter stratified into non-GDM, mild GDM and moderate/severe GDM. We then added the covariates maternal ethnicity, parity and offspring gender to the adjusted models, and maternal height in a final supplementary model. Lastly, we performed the analyses in Europeans and South Asians separately. Estimated mean SD-scores from the adjusted mixed models from each of the four time points were extracted.

### Sample size

The present study was restricted to women of European (n = 379) and South Asian (n = 200) ethnic origin. After excluding twins and those with missing newborn data, OGTT or ultrasound, the final study sample consisted of 349 European and 184 South Asian (63% Pakistani and 37% Sri Lankan/Indian) women (S1 Fig, Flow chart). Of these, 72% were delivered at term and had study-specific measurements at birth.

## Results

### Maternal characteristics

At enrollment (mean gestational week 15), South Asian women were shorter, younger and less educated than European women. Their mean BMI was slightly lower (Table 1 and S2 Fig), but they had more subcutaneous fat represented by thicker skin-folds. Furthermore, South Asian women had significantly higher fasting glucose and HbA1c, with a distribution skewed to the right. Nevertheless, few women had fasting glucose or HbA1c values at levels which may be considered indicative of impaired glucose control, irrespective of later GDM diagnosis (Table 2).

In total 39 (11%) European and 28 (15%) South Asian women were diagnosed with GDM in week 28 according to the WHO 1999 guidelines (Table 2). Relatively more South Asian women had moderate/severe GDM (p = 0.04 for the difference between ethnic groups). Of these, two South Asian and one European woman received insulin treatment during pregnancy. None were treated with oral glucose-lowering drugs.

European women later diagnosed with GDM were more likely to be overweight or obese when entering pregnancy than their non-GDM counterparts, in particular women diagnosed with moderate/severe GDM. However, they showed less weight gain throughout pregnancy than non-GDM women (Table 2). A different pattern was seen in South Asian women diagnosed with GDM, as no differences compared with their non-GDM counterparts were observed for BMI, overweight and gestational weight gain. The majority of South Asian women with moderate/severe GDM (79%) were normal weight and only one was obese.

### Fetal growth

**Fig 1:**
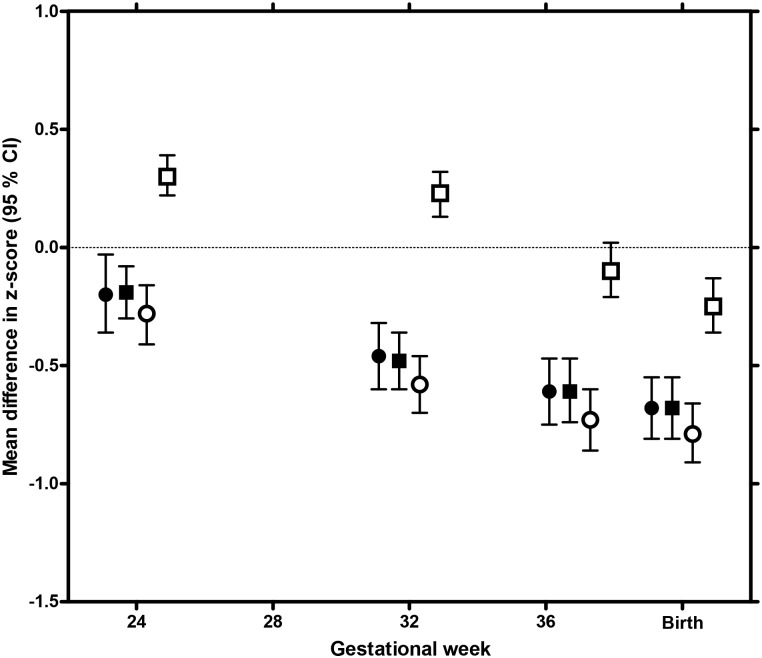
Ethnic differences in fetal size and growth in non-GDM pregnancies. Mean difference in z-score for estimated fetal weight (black circles), head circumference (HC, black squares), abdominal circumference (AC, white circles) and femur length/length (white squares), in South Asian non-GDM, compared with European non-GDM pregnancies, at four time points (gestational week 24, 32 and 37 during pregnancy and at birth). Estimates are gestational age specific z-scores extracted from a linear mixed model, adjusted for maternal parity and fetal gender. Ethnic Europeans are reference group, represented by the zero-line.

In week 24, fetuses of South Asian non-GDM mothers had longer femurs, but smaller ACs than their European counterparts (Fig 1). From this time they grew significantly slower (Fig 1, S1 Table), and at birth they were smaller on all measures (Fig 1).

Fetuses of mothers who were later diagnosed with GDM were smaller on all fetal measures in week 24 (mean difference in EFW z-score: -0.30 SD (-0.53, -0.07), p = 0.01), compared with fetuses of non-GDM mothers. From this time until birth they showed faster growth (S2a Table). However, when we categorized GDM into “mild” and “moderate/severe” GDM, and South Asian and European women were analyzed separately, different patterns emerged (S2b Table).

**Fig 2:**
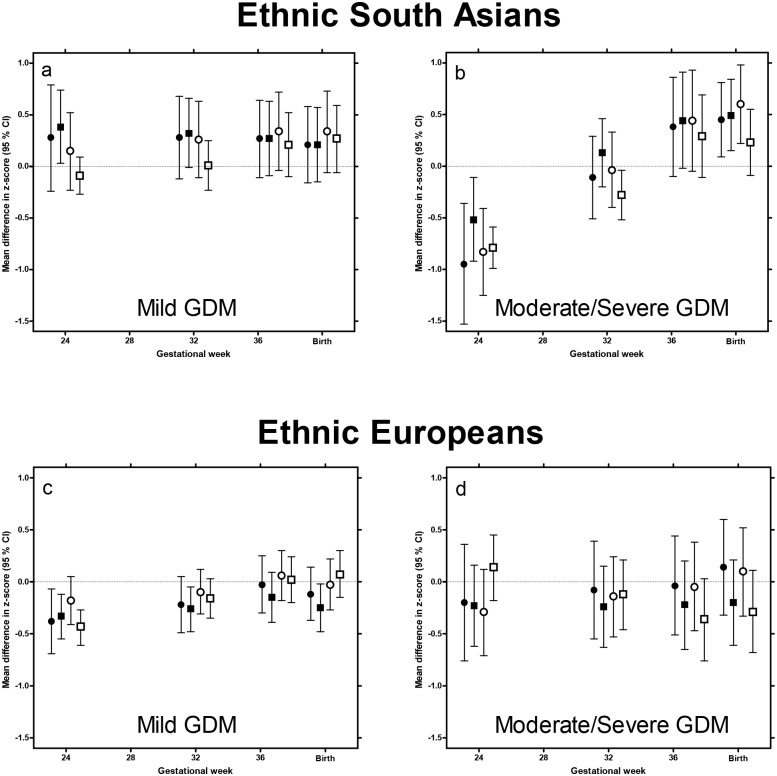
Impact of gestational diabetes on fetal size and growth in European and South Asian pregnancies. Mean difference in z-score for estimated fetal weight (black circles), head circumference (HC, black squares), abdominal circumference (AC, white circles) and femur length/length (white squares), in mothers diagnosed with mild or moderate/severe gestational diabetes (GDM), compared with non-GDM of same ethnicity, at four time points (gestational week 24, 32 and 37 during pregnancy and at birth). Europeans and South Asian were analyzed separately. Fetuses not exposed to GDM constituted the reference group in both ethnicities on all measures, represented by the zero-lines. Estimated differences from the reference groups were extracted from linear mixed models., adjusted for maternal parity and fetal gender.

South Asian fetuses exposed to mild GDM had a similar size and growth rate during the second half of pregnancy as South Asian non-GDM fetuses (Fig 2 and S2b Table). In contrast, fetuses exposed to moderate/severe GDM were markedly smaller on all measures in week 24 (Fig 2). EFW was -0.95 SD (-1.53, -0.36), p<0.001) at this time point. From this time until birth, they showed a faster growth rate on all measures, and birth weight was 0.45 SD ((0.09, 0.81), p = 0.01) higher than in non-GDM pregnancies. This was also reflected in a 92 g larger placenta weight (p<0.05) (Table 3).

**Table 3:** Pregnancy, birth and neonatal outcomes by ethnic origin and GDM-status; mild or moderate/severe GDM, compared with non-GDM women.

		Europe			South Asia	
	Non-GDM	Mild	Moderate/Severe	Non-GDM	Mild	Moderate/severe
	n = 310	n = 30	n = 9	n = 156	n = 14	n = 14
Pregnancy and birth complications						
Mild hypertension/preeclampsia	20 (7%)	3 (10%)	0 (0)	5 (3%)	1 (7%)	2 (14%)
Severe hypertension/preeclampsia^a^	2 (1%)	2 (7%)	0 (0)	3 (2%)	0 (0)	1 (7%)
Spontaneous start of birth	240 (79%)	23 (77%)	3 (33%)***	132 (85%)	8 (57%)	4 (28)***
Non-operative vaginal delivery	221 (71%)	18 (60%)	6 (67%)	118 (76%)	8 (57%)	9 (64%)
Composite, 4 birth complications^b^	90 (29%)	10 (33%)	3 (33%)	52 (33%)	6 (43%)	4 (29%)
Neonatal outcomes						
Gestational age, days, mean (95%CI)	281 (280, 282)	279 (275, 283)	278 (270, 285)	277 (275, 279)	274 (267, 281)	278 (272, 285)
Preterm delivery (<37 weeks)	13 (4%)	4 (13%)	1(11%)	11 (7%)	2 (15%)	0 (0)
Placenta weight, g, mean (95% CI)	691 (674, 708)	714 (659, 769)	731 (614, 849)	614 (591, 638)	625 (550, 702)	706 (627, 785)*
Gender boy	159 (53%)	16 (53%)	3 (33%)	82 (53%)	7 (54%)	8 (57%)
Birth weight, g, mean (95% CI)	3581 (3523, 3640)	3496 (3307, 3885)	3647 (3302, 3993)	3209 (3128, 3290)	3252 (2972, 3531)	3412 (3143, 3682)
Birth weight z-score, mean (95% CI)^c^	0.04 (-0.07, 0.14)	-0.05 (-0.39, 0.29)	0.41 (-0.22, 1.03)	-0.63 (-0.77, -0.49)	-0.38 (-0.86, 0.10)	-0.25 (-0.71, 0.22)
Small for gestational age (SGA)^c^	26 (8%)	4 (13%)	0 (0)	41 (26%)	0 (0)	3 (21%)
Large for gestational age (LGA)^c^	29 (9%)	5 (17%)	2 (22%)	4 (3%)	0 (0)	1 (7%)

Differences between each GDM group, compared with non-GDM women, within ethnic group, were assessed using chi-square tests for categorical and t-tests for continuous variables. Numbers are n (%) or mean (95% CI).

* Indicates a significant difference from non-GDM women within ethnic group of p≤0.05

** Indicates a significant difference from non-GDM women within ethnic group of p<0.01

*** Indicates a significant difference from non-GDM women within ethnic group of p<0.001

^a^ Categorized at birth, from hospital records, defined as preeclampsia or severe hypertension with large clinical implications (i.e. induction of birth), preeclampsia before gestational week 34, eclampsia or HELLP-syndrome.

^b^ The presence of one or more of four birth complications; acute cesarean section, meconium-stained amniotic fluid, grade 3–4 perineal tear or Apgar score <8 at 1 minute.

^c^ By Norwegian national birth weight references, based on all births in Norway from 1967–1998.

European fetuses exposed to GDM also tended to be smaller in week 24 and show faster growth than their non-GDM counterparts (Fig 2 and S2a and S2b Table).

## Discussion

In this study we observed that fetuses of mothers later diagnosed with GDM tended to be smaller in week 24, compared with fetuses of non-GDM mothers. From this time until birth they showed a faster growth. This pattern was most pronounced in South Asian fetuses of mothers diagnosed with moderate/severe GDM, who were the smallest for all body measures in mid pregnancy.

This study is unique, as we were able to combine longitudinal maternal and offspring data from early pregnancy to birth, including GDM-data from universal screening, in a well-characterized population-based cohort, in two different ethnic groups. The smaller fetal size in mid-pregnancy in GDM, compared with non-GDM pregnancies, was unexpected. However, an early fetal growth delay has been reported both in Type 1 and Type 2 diabetes [18, 19]. There are only few reports of fetal growth in pregnancies which are later diagnosed with GDM. A recent study from the UK did not observe a significant association between GDM and fetal size in week 20, in a predominantly ethnic white British cohort [20]. They did, however, find an accelerated fetal growth velocity between week 20 and week 28, hence preceding the clinical diagnosis of GDM, which is in line with our study.

The slower growth in early pregnancy in Type 1 and Type 2 diabetes has been associated with indicators of poor glucose control in early pregnancy and with lower levels of some biomarkers indicating impaired early placentation [21]. Few women in our study had high HbA1c or high fasting blood glucose in early pregnancy, irrespective of GDM-severity later in pregnancy. For a given BMI, South Asians tend to have lower lean mass, including lower muscle mass and fewer insulin-producing β-cells, but relatively more fat mass than ethnic Europeans [3, 22, 23]. We have previously shown from the same cohort that South Asian mothers had more subcutaneous fat and higher serum-leptin levels in early pregnancy compared with Europeans, despite having a lower BMI, indicating a thin-fat phenotype.[24]. Hence, the substantial “growth delay” in mid pregnancy, observed in fetuses of predominantly normal weight South Asian women diagnosed later with moderate/severe GDM, could still indicate subtle metabolic and placental abnormalities from an early stage in pregnancy.

Our results could also indicate that there may be different types of GDM. One being GDM in an obese women, with pre-existing insulin resistance and a sufficient capacity to produce insulin in a non-pregnant situation, but decreased capacity to compensate for the added insulin resistance caused by pregnancy. The other being GDM in a “thin-fat” woman, predominantly due to a reduced capacity to produce insulin but also diminished insulin sensitivity due reduced muscle mass [25].

It has also been speculated that maternal hyperglycemia during the second half of pregnancy may in part be triggered by the fetus through the placenta, to promote intrauterine “catch-up” after early growth failure [26]. If such a hyperglycemic response is aiming to compensate for early placental insufficiency, we could further speculate that very intensive treatment of GDM, primarily by a very strict glucose control, if followed by a low maternal weight gain, may aggravate growth failure, in particular in normal weight GDM women.

The key challenge is that optimal fetal growth remains to be defined. Customized fetal growth charts aim to predict the growth potential, i.e. “optimal growth” for each baby, by adjusting for characteristics which influence birth weight, such as maternal height, parity and ethnic origin, and by excluding pathological factors such as smoking and diabetes. However, ethnic differences in fetal growth are observed, and only partly explained by key maternal factors, such as parity, socioeconomic position and height [12, 27]. Factors which are independently associate with fetal size at different time points during pregnancy, and across ethnic groups, await elucidation [6, 28]. Fetuses of South Asian non-GDM mothers had longer femurs, but smaller ACs in mid pregnancy, compared with their European counterparts. From this time until birth they showed a slower growth on all measures. Hence, at birth South Asian neonates were thinner, represented by a markedly smaller AC, while length and HC were modestly smaller. Whether this growth pattern is optimal for South Asians is not known. When using the Norwegian national birth weight references, more than 20% of the South Asian neonates in our cohort were defined as being small for gestational age, while few were large for gestational age, reflecting that the birth weight distribution is skewed compared with the distribution in the native Norwegian population. We did not observe any differences in birth complications between pregnancies with and without GDM, or between ethnic groups in our study. However, we have limited statistical power to answer these comparisons. In the short run, for perinatal outcomes, growth restriction diagnosed by ethnic-specific fetal growth charts seems to better predict adverse perinatal outcomes [29]. Nevertheless, for long term outcomes, such as adiposity and type 2 diabetes, both “thinness” at birth and excess fetal growth have been associated with a higher risk [1]. From this we could speculate that South Asian fetuses, both those exposed to GDM and those not, may be at increased risk of type 2 diabetes in later life.

The study has some limitations. Most women diagnosed with GDM were treated non-pharmacologically, indicating that there were few cases of severe GDM. However, treatment could still have influenced the fetal growth patterns. Furthermore, the limited number of GDM-cases, further categorized into two groups, primarily based on elevated 2-hour-values, restricts our power to adjust for many covariates, and to explore interactions. However, as women in the two GDM-categories were given different follow-up after being diagnosed, the alternative of not taking this into account could cause bias. Almost all South Asian women with moderate/severe GDM were normal weight before entering pregnancy but had similar weight gain as non-GDM women. Most Europeans, however, were obese and had substantially less weight gain than their non-GDM counterparts. Adjusting for these factors could therefore potentially also cause bias. Our findings illustrate the complexity involved in the relationship between maternal factors and fetal growth, should be interpreted with caution.

### Conclusions

To conclude, we observed differences in fetal size and body proportions from before diagnosis and treatment of glucose intolerance. The most prominent growth deviations was observed in fetuses of South Asian mothers diagnosed with moderate/severe GDM. Their babies were small in size in mid-pregnancy but but subsequently grew faster until birth compared with babies of non-GDM mothers. The mechanisms underlying these differences are likely to be at least partly present before the pregnancy. Our results highlights the need for large-scale comparative studies of serial fetal growth from early pregnancy. This could have implications for the timing of diagnosis and the treatment of pregnancy-related hyperglycemia in different ethnic groups.

## Supporting information

S1 Fig Flow chart showing maternal-fetal-pairs selected for analysis. (PDF)

S2 Fig Distribution of pre-pregnant BMI and HbA1c levels. (PDF)

S1 Table Ethnic differences in fetal growth rate in non-GDM pregnancies. (DOC)

S2 Table Differences in fetal growth velocity in GDM compared with non-GDM. (DOCX)
