# Supplementary figures and images for: Fetal growth trajectories in pregnancies of European and South Asian mothers with and without gestational diabetes, a population-based cohort study
Source: PLoS One. 2017 Mar 2;12(3):e0172946. doi: 10.1371/journal.pone.0172946 (PMC5333847; doi:10.1371/journal.pone.0172946)

**S1 Fig.** Flow chart showing maternal-fetal-pairs selected for analysis.

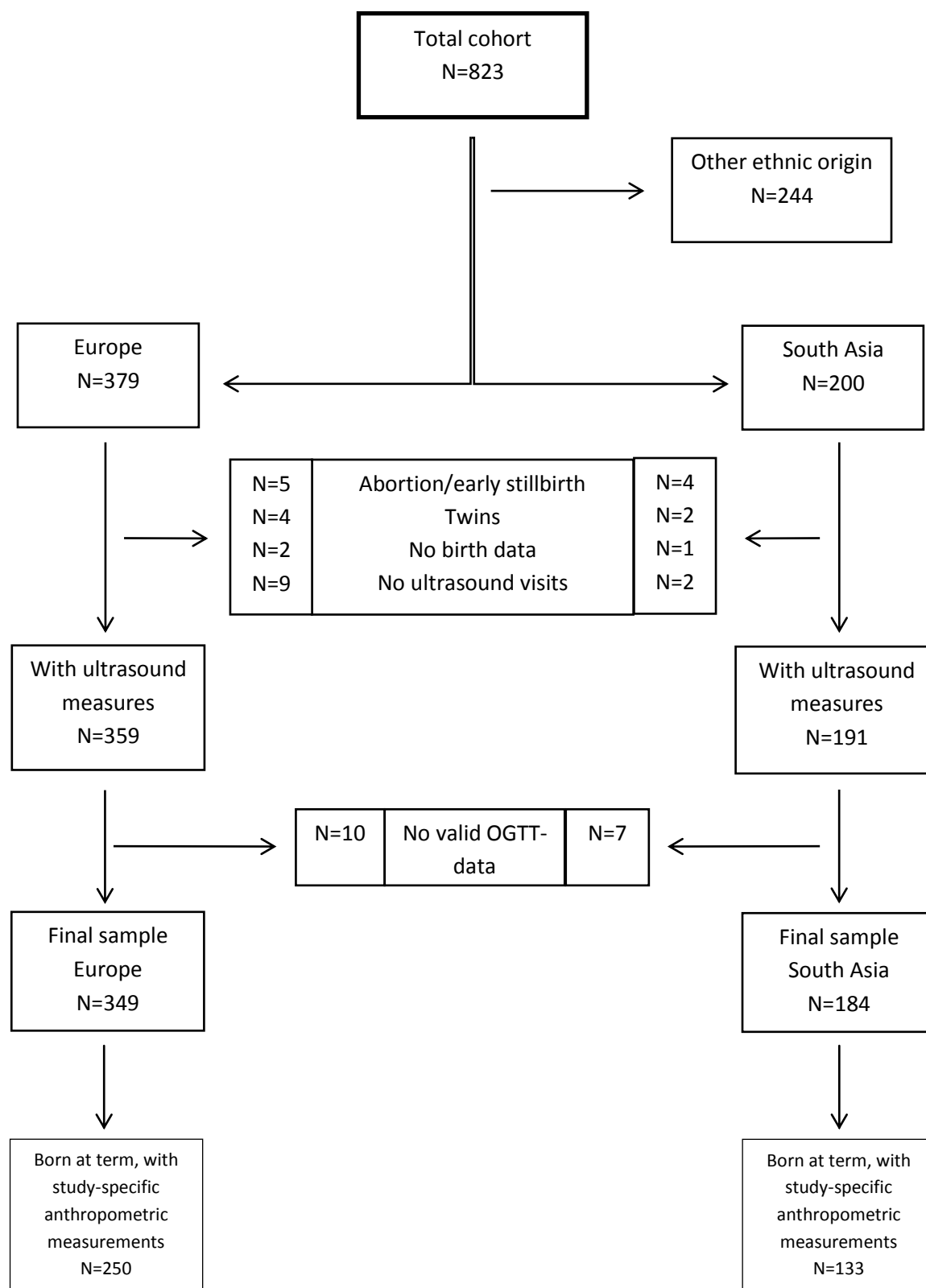

Supplement: S1 Fig — (PDF) [file pone.0172946.s001.pdf]
