# Supplementary material for: Fetal growth trajectories in pregnancies of European and South Asian mothers with and without gestational diabetes, a population-based cohort study
Source: PLoS One. 2017 Mar 2;12(3):e0172946. doi: 10.1371/journal.pone.0172946 (PMC5333847; doi:10.1371/journal.pone.0172946)

S2 Fig. Distribution of pre-pregnant BMI and HbA1c level in European (black bars, solid line) and South Asians (white bars, dotted line), with non-linear robust fitted Gaussian curves.

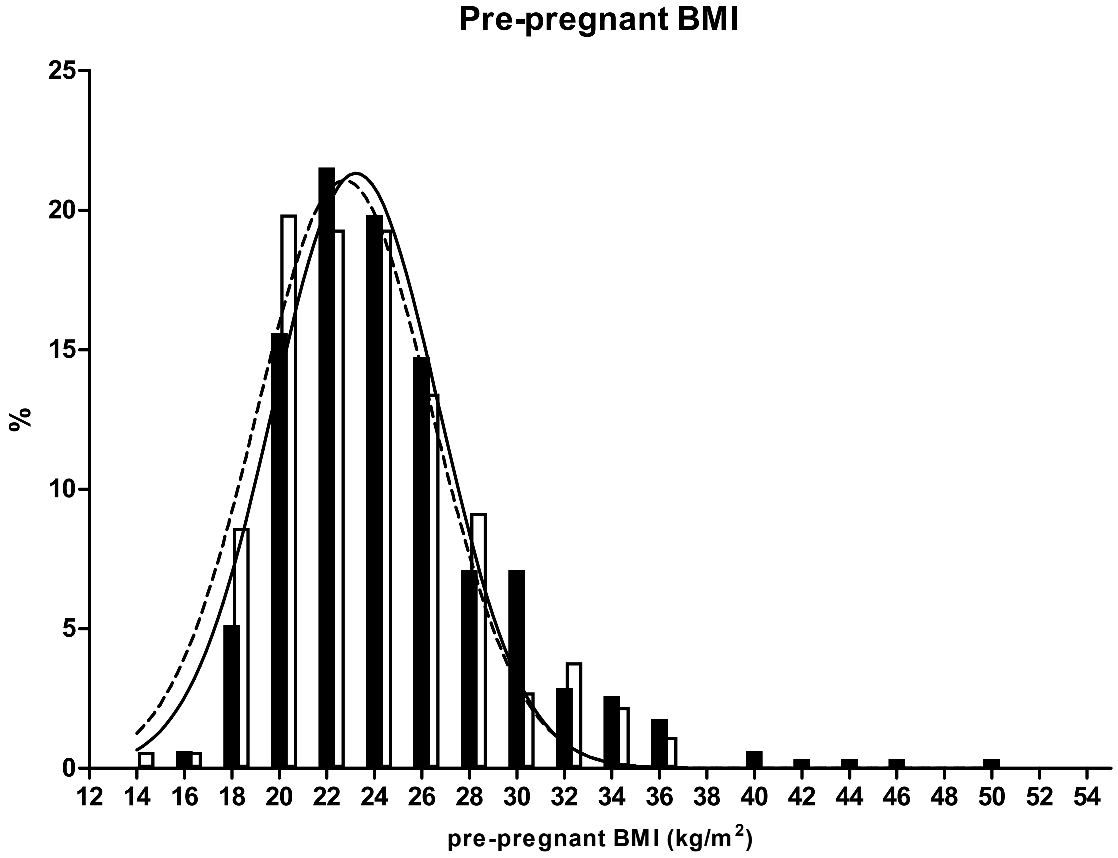

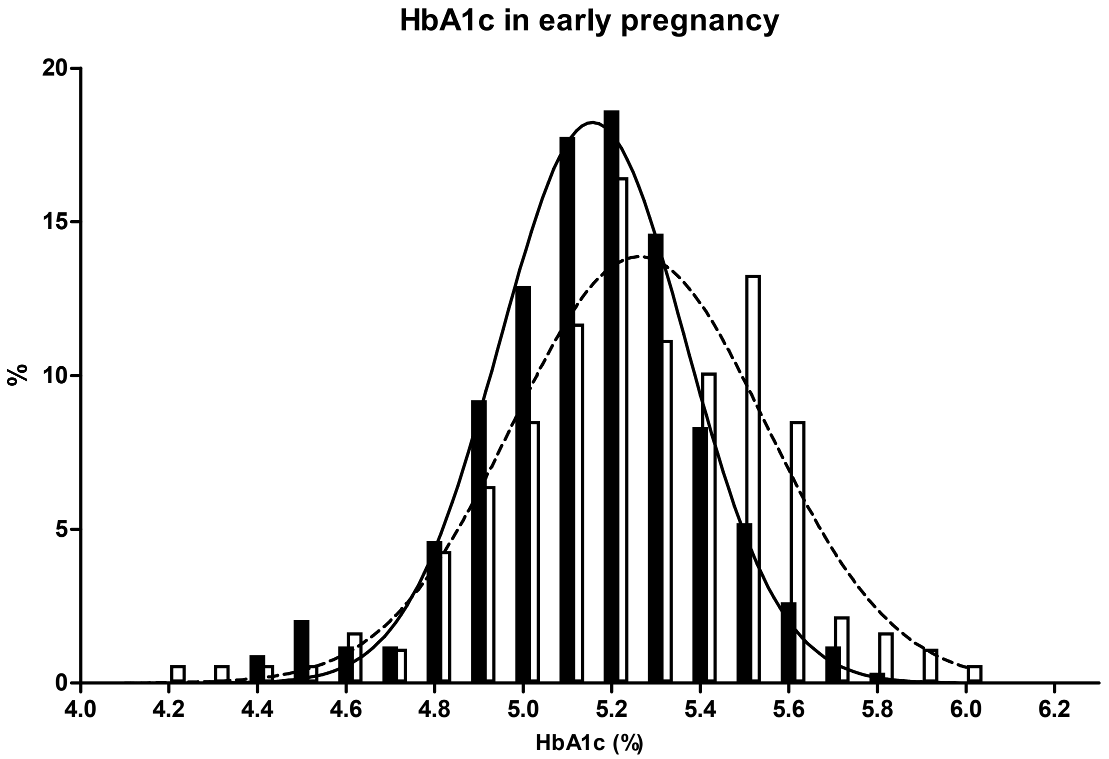

Supplement: S2 Fig — (PDF) [file pone.0172946.s002.pdf]
