# Supplementary material for: Fetal growth trajectories in pregnancies of European and South Asian mothers with and without gestational diabetes, a population-based cohort study
Source: PLoS One. 2017 Mar 2;12(3):e0172946. doi: 10.1371/journal.pone.0172946 (PMC5333847; doi:10.1371/journal.pone.0172946)
Supplement: S1 Table — (DOC) [file pone.0172946.s003.doc]

**S1 Table. Ethnic differences in fetal growth rate in South Asian non-GDM compared with ethnic European non-GDM pregnancies.**

|  |  | Model 0 |  |  | Model 1 |  |  | Model 2 |  |
| --- | --- | --- | --- | --- | --- | --- | --- | --- | --- |
|  |  | β (95 % CI) | p |  | β (95 % CI) | p |  | β (95 % CI) | p |
| Weight |  | -0.025 (-0.038, -0.012) | <0.001 |  | -0.028 (-0.041, -0.015) | <0.001 |  | -0.016 (-0.031, -0.001) | 0.03 |
| Head circumference |  | -0.028 (-0.042, -0.014) | <0.001 |  | -0.030 (-0.044, -0.016) | <0.001 |  | -0.021 (-0.037, -0.005) | 0.01 |
| Abdominal circumference |  | -0.029 (-0.042, -0.016) | <0.001 |  | -0.032 (-0.045, -0.019) | <0.001 |  | -0.024 (-0.039, -0.008) | 0.003 |
| Length |  | -0.037 (-0.052, -0.022) | <0.001 |  | -0.038 (-0.053, -0.023) | <0.001 |  | -0.027 (-0.044, -0.009) | 0.003 |

The βs are the estimates for the interaction terms between gestational age (weeks) and ethnicity, from four separate Linear Mixed Model, using estimated fetal weight, head circumference, abdominal circumference and length as outcomes respectively. The βs indicates how many SD South Asian fetuses increase or decrease in size per week gestation, compared with European fetuses. Negative βs indicate a slower fetal growth rate.

Model 0: Only adjusted for ethnicity, gestational age (GA), GA^2^ and GA*ethnicity.

Model 1: Also adjusted for gender, parity and GA*parity.

Model 2: Also adjusted for maternal height and GA*height.
