# Supplementary material for: Fetal growth trajectories in pregnancies of European and South Asian mothers with and without gestational diabetes, a population-based cohort study
Source: PLoS One. 2017 Mar 2;12(3):e0172946. doi: 10.1371/journal.pone.0172946 (PMC5333847; doi:10.1371/journal.pone.0172946)
Supplement: S2 Table — (DOCX) [file pone.0172946.s004.docx]

**S2 Table (a). Differences in fetal growth velocity in GDM compared with non-GDM pregnancies**

|  |  | Model 0 |  |  | Model 1 |  |  | Model 2 |  |
| --- | --- | --- | --- | --- | --- | --- | --- | --- | --- |
|  | | β (95 % CI) | p |  | β (95 % CI) | p |  | β (95 % CI) | p |
|  | Weight | 0.021 (0.003, 0.039) | 0.02 |  | 0.025 (0.008, 0.043) | 0.005 |  | 0.030 (0.012, 0.048) | 0.001 |
|  | HC | 0.006 (-0.013, 0.020) | 0.5 |  | 0.010 (-0.0.009, 0.030) | 0.3 |  | 0.014 (-0.006, 0.033) | 0.2 |
|  | AC | 0.023 (0.005, 0.042) | 0.01 |  | 0.027 (0.008, 0.045) | 0.004 |  | 0.031 (0.012,0.049) | 0.001 |
|  | Length | 0.022 (0.002, 0.043) | 0.04 |  | 0.025 (0.005, 0.046) | 0.02 |  | 0.029 (0.008, 0.050) | 0.006 |
| Differences in fetal growth velocity expressed as the β for the interaction term between the GDM and gestational age (weeks). Numbers are calculated from four Linear Mixed Models, using weight, head circumference (HC), abdominal circumference (AC) and length measured through four time points during the second half of pregnancy until birth as outcomes respectively. The βs indicate how many SD fetuses of GDM mothers increase in size, in comparison with fetuses of non-GDM mothers, per week gestation. | | | | | | | | | |

Model 0, only adjusted for gestational age (GA) and GA^2^

Model 1, also adjusted for offspring gender and maternal parity and ethnic origin

Model 2, also adjusted for maternal height

**S2 Table (b). Differences in fetal growth rate in mild GDM and moderate/severe GDM, compared with non-GDM pregnancies**

|  |  |  |  |  | Analyses stratified by ethnicity | | | | |
| --- | --- | --- | --- | --- | --- | --- | --- | --- | --- |
|  |  | Model 3 |  |  | Model 3 South Asia |  |  | Model 3 Europe |  |
|  |  | β (95 % CI) | p |  | β (95 % CI) | p |  | β (95 % CI) | p |
| Weight |  |  |  |  |  |  |  |  |  |
|  | Mild GDM | 0.016 (-0.005, 0.037) | 0.1 |  | -0.004 (-0.046, 0.038) | 0.9 |  | 0.023 (-0.001, 0.047) | 0.06 |
|  | Moderate/severe GDM | 0.061 (0.031, 0.091) | <0.001 |  | 0.081 (0.037, 0.125) | <0.001 |  | 0.032 (-0.011, 0.075) | 0.2 |
| HC |  |  |  |  |  |  |  |  |  |
|  | Mild GDM | 0.002 (-0.021, 0.025) | 0.9 |  | -0.008 (-0.054, 0.039) | 0.7 |  | 0.006 (-0.019, 0.032) | 0.6 |
|  | Moderate/severe GDM | 0.038 (0.006, 0.071) | 0.02 |  | 0.058 (0.010, 0.106) | 0.02 |  | 0.012 (-0.037, 0.060) | 0.6 |
| AC |  |  |  |  |  |  |  |  |  |
|  | Mild GDM | 0.014 (-0.008, 0.036) | 0.2 |  | 0.011 (-0.031, 0.054) | 0.6 |  | 0.014 (-0.011, 0.040) | 0.3 |
|  | Moderate/severe GDM | 0.065 (0.034, 0.096) | <0.001 |  | 0.092 (0.049, 0.134) | <0.001 |  | 0.031 (-0.015, 0.077) | 0.2 |
| Length |  |  |  |  |  |  |  |  |  |
|  | Mild GDM | 0.029 (0.005, 0.054) | 0.02 |  | 0.018 (-0.029, 0.066) | 0.5 |  | 0.033 (0.004, 0.061) | 0.03 |
|  | Moderate/severe GDM | 0.030 (-0.006, 0.066) | 0.1 |  | 0.071 (0.021, 0.122) | 0.006 |  | -0.021 (-0.074, 0.031) | 0.4 |

Differences in fetal growth velocity are expressed as the β for the interaction term between the GDM and gestational age (weeks). Numbers are calculated from four Linear Mixed Models, using weight, head circumference (HC), abdominal circumference (AC) and length measured through four time points during the second half of pregnancy until birth as outcomes respectively. The βs indicate how many SD fetuses of GDM mothers increase in size, compared with fetuses of non-GDM mothers, per week gestation.

All models are adjusted for fetal gender and maternal ethnicity, parity and height.
